# Predictors of foot pain in the community: the North West Adelaide health study

**DOI:** 10.1186/s13047-016-0150-9

**Published:** 2016-07-13

**Authors:** Tiffany K. Gill, Hylton B. Menz, Karl B. Landorf, John B. Arnold, Anne W. Taylor, Catherine L. Hill

**Affiliations:** School of Medicine, Faculty of Health Sciences, The University of Adelaide, Level 7, SAHMRI, North Tce, Adelaide, SA 5000 Australia; School of Allied Health, College of Science, Health and Engineering, La Trobe University, Bundoora, VIC 3083 Australia; Melbourne Health, Allied Health Department, 300 Grattan Street, Parkville, VIC 3050 Australia; Alliance for Research in Exercise, Nutrition and Activity (ARENA), Sansom Institute for Health Research, School of Health Sciences, University of South Australia, Adelaide, SA 5000 Australia; Population Research and Outcome Studies, Discipline of Medicine, Faculty of Health Sciences, The University of Adelaide, Adelaide, SA 5005 Australia; Rheumatology Unit, The Queen Elizabeth Hospital, Woodville Rd, Woodville, SA 5011 Australia; School of Medicine, The University of Adelaide, Adelaide, SA 5005 Australia

**Keywords:** Pain, Epidemiology, Predictors, Cohort, Population studies

## Abstract

**Background:**

Foot pain has been shown to be prevalent across all age groups. The presence of foot pain may reduce mobility and impact on the ability to undertake activities of daily living. The aim of this study was to determine factors that are predictive of foot pain in a community based sample of the general population.

**Methods:**

This study analysed data from the North West Adelaide Health Study, a cohort study located in the northwestern suburbs of Adelaide, South Australia. Data were obtained between 2004–2006 and 2008–2010, using a self-completed questionnaire, computer assisted telephone interviewing, and a clinical assessment. The sensitivity, specificity and positive predictive values of variables were determined and generalised linear models ascertained the variables associated with the highest relative risk of self-reporting foot pain in 2008–2010 based on the data obtained in 2004–2006.

**Results:**

The prevalence of foot pain in 2004–2006 was 14.9 % (95 % CI 13.6–16.4) and in 2008–2010, 29.9 % (95 % CI 27.5–32.5). Variables with the highest sensitivity were: female sex, ever having back pain, self-reported arthritis, body mass index (BMI) classified as obese and having foot pain in 2004–2006, while most variables demonstrated high specificity. Those with the highest risk of reporting foot pain in 2008–2010 were those with depressive symptoms, self-reported arthritis, high BMI, self-reported upper limb pain and foot pain (in general or in specific regions of the foot) in 2004–2006.

**Conclusion:**

Foot pain is common in the general population and those with the greatest risk of foot pain potentially represent a high level of chronicity and potential burden on the health system. Addressing the factors that predict foot pain, as well as the provision of targeted messages to highlight the importance of managing foot pain, may help reduce the impact on the population.

## Background

Foot pain has long been recognised as being widespread in older people, affecting approximately one in three people aged over 65 years [1, 2]. However, foot pain has also been shown to be highly prevalent among younger adults [3]. A recent systematic review demonstrated that 24 % of community dwelling participants aged 45 years and over reported frequent foot pain (pain on most days) [4].

Several potential risk factors for foot pain have been identified from cross-sectional studies, including obesity [5], osteoarthritis [6], diabetes [7], and depression [8]. Alcohol consumption and smoking may both have an impact on the feet, although few studies have directly explored this. Alcohol consumption has been shown to be associated with single or multi-site pain in men [9] and also with chronic widespread pain in both men and women [10]. Smoking has been shown to generally have a negative effect on the musculoskeletal system particularly with regard to bone mineral density [11], although variable associations have been shown with regard to smoking and the presence of osteoarthritis [12].

Physical inactivity has been identified as a risk factor for chronic musculoskeletal pain [13] and it is also recognised that activity has a beneficial effect on pain and disability [14]. However, it is also possible that those with musculoskeletal pain are less likely to undertake activity [15], particularly if pain is present in the foot. Arthritis is also a factor associated with foot pain, in particular rheumatoid arthritis and osteoarthritis of the midfoot and/or the first metatarsophalangeal joint [16]. In fact, over 35 % in a sample of patients with rheumatoid arthritis identified that foot pain was the presenting symptom [17]. It is of note, that pain in multiple sites has been shown by Keenan et al. [18] to be more common than joint pain at a single site. Gill et al. [19] have also shown previously that incident, recurrent and resolved shoulder pain were all associated with self-reported pain in various other joints.

Although these findings suggest a link between a range of possible risk factors and foot pain, their cross-sectional study designs cannot infer causation. Therefore, the aim of this study was to determine the predictive value of these variables, with regard to future foot pain by utilising prospective cohort data obtained from the North West Adelaide Health Study in 2004–2006 and 2008–2010.

## Methods

The North West Adelaide Health Study (NWAHS) is a representative longitudinal study of over 4000 randomly selected adults aged 18 years and over at the time of recruitment from the north-west region of Adelaide, South Australia. The sample region represents approximately half of the metropolitan area (total population of approximately 1.2 million) and almost one-third of the population in South Australia (population of approximately 1.6 million), which has the second highest population of older people among all of the Australian states and territories [20]. The aim of the study is to provide self-reported data that has been longitudinally measured to inform strategies and policies to prevent, detect and manage a range of chronic conditions [21]. The study commenced with Stage 1, in 1999 to 2003, Stage 2 was conducted between 2004 and 2006 and Stage 3 was conducted between 2008 and 2010. Ethical approval for each stage of the study was obtained from the Human Research Ethics committee of The Queen Elizabeth Hospital, Adelaide, South Australia and all participants provided written informed consent.

### Data collection

Participant information was obtained from a Computer Assisted Telephone Interview (CATI), a self-complete questionnaire and a clinical assessment at each stage [21, 22]. The original cohort of participants was 4056, with 3205 (81.5 % of the eligible sample) participating in all three data collections (the CATI survey, self-complete questionnaire and clinical assessment) in Stage 2 and 2487 (67.0 % of the eligible sample) completing these assessments in Stage 3. Foot pain related data were collected in Stage 2 and Stage 3, and these stages are the focus of this study.

### Stage 2 variables

In 2004–2006, smoking, physical activity and alcohol consumption were determined from the responses to the self-completed questionnaire. The level of physical activity was determined from descriptions of physical activity type and time over a 2 week time frame [23]. Smoking was determined using standard questions relating current smoking and the frequency and alcohol consumption was determined from questions based on the National Heart Foundation Risk Factor Prevalence Study undertaken in 1989 [24].

Depression was determined from the CATI response to the Centre for Epidemiological Studies in Epidemiology Depression questionnaire (CES-D) [25]. Participants were asked if they been told by a doctor that they had arthritis. If the response was in the affirmative, participants were then asked what type of arthritis they had. Participants were also asked if they had used podiatry services in the last 12 months. The presence of pain and/or stiffness in the shoulder, hip, knee and back was determined by asking participants if they had ever had pain/stiffness in those areas on most days for at least a month. The presence of hand pain was determined by asking if participants had had pain, aching or stiffness on most days for at least a month.

The presence of diabetes was determined from a self-reported doctor diagnosis of diabetes and/or a fasting plasma glucose level of greater than or equal to 7.0 mmol/L. Age was calculated from the participants’ date of birth and the date participants attended their clinical assessment. During the clinical assessment height and weight were measured with standardised protocols. A wall mounted stadiometer measured height to the nearest 0.5 cm and weight was measured using calibrated scales to the nearest 0.1 kg. BMI was then calculated using the following formula [26]: weight (kg)/height (m^2^).

### Foot pain

In 2004–2006, participants were asked as part of the telephone questionnaire, “On most days, do you have pain, aching or stiffness in either of your feet?" In addition, as part of the clinical assessment, which included measurements such as blood pressure, lung function and taking of blood samples, participants were shown a diagram of the foot and asked to identify areas that they had pain, that is, hindfoot, forefoot, toes, nails, arch, ball and heel. The diagram was based on the Framingham Foot Study, 2002–2008 [27]. In 2008–2010, participants were asked as part of the self-complete questionnaire “Over the past month, have you had pain, aching or stiffness in either of your feet on most days?” This question was used to more specifically determine the presence of current pain.

### Data weighting

In Stage 1, data were weighted by region (western and northern health regions), age group, sex and probability of selection in the household to the Australian Bureau of Statistics 1999 Estimated Resident Population and the 2001 Census data. Weighting was undertaken to reflect the population of interest and to correct for potential non-response bias in which some groups of respondents may be over- or under-represented. Weighting variables were also created for Stage 2 and 3 using the 2004 and 2009 Estimated Resident Population for South Australia respectively, and also incorporating participation in the three components (CATI, self-complete questionnaire, clinic), whilst retaining the original weight from Stage 1 in the calculation. All analyses in this paper, where applicable, are weighted to the population of the northern and western suburbs of Adelaide.

### Statistical analyses

Statistical analyses were conducted using STATA version 13 (StataCorp, College Station, TX, USA). The weighted prevalence (using the “svy” estimators and “pweight” as the weighting variable) of foot pain obtained from Stage 2 and Stage 3 was determined and the frequency of each of the variables measured as part of the Stage 2 assessment, for those with and without foot pain on most days in the last month, was determined. These variables were then used as predictors for the presence of foot pain as determined at Stage 3 and thus, tests aimed at examining how well variables predicted the presence of foot pain were used. The sensitivity (probability that the associated factor is present when foot pain is present), specificity (probability that the associated factor is not present when foot pain is not present), positive predictive value of each of the predictor variables (the probability that foot pain is present given that the associated factor is present) and the area under the Receiver Operating Characteristic (ROC) curve (the summary of the performance of each variable in terms of the ability to predict foot pain) were all determined. An area under the ROC curve equal to one indicates that a variable perfectly predicts those at risk of the outcome whereas a value of 0.5 represents a predictor that is no better than chance at predicting the outcome of interest. The area under the ROC curve was calculated using predictors as binary variables as this is the most common occurrence in clinical practice, however is must be noted that generally continuous variables are more appropriate to use for ease of interpretability.

Generalised linear models using the binary outcome variable of presence of foot pain/no foot pain on most days in the past month were used with the “svy” estimators and weighted data to determine the relative risks (RR) of each of the predictors in association with the outcome variable. Multivariable models were created with all relevant predictor variables included. The participants reporting toe and nail pain were combined (due to the small number of participants reporting the presence of toe pain). In addition, the areas of joint pain were combined. Hand and shoulder pain were defined as upper limb pain and hip and knee pain were defined as lower limb pain. Back pain remained a separate variable. In total, four separate multivariable models were created with adjustment for all possible predictors.

## Results

The prevalence of foot pain in 2004–2006 was 14.9 % (95 % CI 13.6–16.4) and 29.9 % (95 % CI 27.5–32.5) in 2008–2010. Table 1 shows the proportion of those with foot pain in the last month by each of the 2004–2006 characteristics. In particular, foot pain was higher among females, older people, those with symptoms of depression and those categorised as obese.

**Table 1:** Predictor variables for those without and with foot pain on most days in the last month (2008–2010) ^a^

	Without foot pain		With foot pain	
	Number	Percent	Number	Percent
Sex				
Male	915	73.9	323	26.1
Female	858	66.4	435	33.6
Age group				
20–34 years	509	74.7	172	25.3
35–44 years	416	76.8	118	23.2
45–54 years	304	66.3	164	33.7
55–64 years	225	65.3	133	34.7
65–74 years	160	62.0	98	38.1
75 years and over	101	62.6	59	37.4
Smoking				
Non smoker	778	72.2	299	27.8
Ex smoker	543	70.8	224	29.2
Current smoker	278	64.2	155	35.8
Depression				
No depression	1560	73.6	561	26.4
Depressive symptoms	141	47.3	157	52.7
Alcohol risk				
Non drinker	786	71.4	316	28.6
Low risk	676	69.1	302	30.9
Intermediate to high risk	72	62.2	44	37.8
Physical activity				
Sedentary	377	65.9	196	34.2
Undertakes some activity	1092	72.0	425	28.2
BMI				
Underweight	21	89.9	2	10.2
Normal	538	76.4	166	23.6
Overweight	639	73.1	235	26.9
Obese	384	59.0	267	41.0
Diabetes				
No	1501	70.8	620	29.2
Yes	96	62.4	57	37.6
Arthritis				
No	1455	75.2	479	24.8
Yes	239	50.4	236	49.6
Foot pain on most days				
No	1583	76.3	493	23.7
Yes	117	34.2	225	65.8
Heel pain				
No	1563	71.7	617	28.3
Yes	26	32.8	53	67.2
Ball pain				
No	1563	72.0	607	28.0
Yes	26	28.8	63	71.2
Arch pain				
No	1553	71.8	610	28.2
Yes	36	37.3	60	62.7
Toe pain				
No	1556	72.0	604	28.0
Yes	33	33.2	65	66.8
Forefoot pain				
No	1542	72.5	584	27.5
Yes	47	35.3	86	64.7
Nail pain				
No	1583	70.3	668	29.7
Yes	5	69.2	2	30.8
Hind foot pain				
No	1550	71.9	605	28.1
Yes	38	36.8	65	63.2
Podiatry use				
No	1590	72.0	619	28.0
Yes	112	52.6	101	47.4
Hip pain/stiffness				
No	1570	72.9	585	27.2
Yes	101	46.9	114	53.1
Back pain/stiffness				
No	1231	76.5	379	23.5
Yes	471	58.2	338	41.8
Hand pain/stiffness				
No	1531	73.2	561	26.8
Yes	167	51.9	155	48.1
Shoulder pain/stiffness				
No	1409	75.5	457	24.5
Yes	289	52.7	259	47.3
Knee pain/stiffness				
No	1481	73.5	533	26.5
Yes	203	53.3	177	46.7

^a^ All don’t know/not stated responses removed from the analysis. The weighting of data may lead to rounding discrepancies and totals not adding up

Table 2 shows the sensitivity, specificity, positive predictive value and area under the ROC (95 % CI) curve for the presence of foot pain in 2008–2010 using each of the predictor variables from 2004 to 2006. Those with the highest sensitivity, that is, the ability to correctly identify those with foot pain were: sex (female), self-reported arthritis, BMI classified as obese, ever having had back pain/stiffness and having reported foot pain in 2004–2006. The variable with the lowest specificity was sex (female) while all other variables were able to strongly identify those without foot pain. In general, foot pain and the specific areas of foot pain all had the highest positive predictive values. Values of the area under the ROC curve were all similar, indicating that most of the variables were no better than chance at predicting foot pain. Those with the highest value were general foot pain and self-reported arthritis. However, it is also of note that these were the variables with the highest positive predictive value, that is, those factors most likely to be present when foot pain it present.

**Table 2:** Sensitivity, specificity, positive predictive value, area under ROC curve of predictor variables associated with the presence of foot pain on most days in the last month

	Sensitivity (%)	Specificity (%)	Positive predictive value (%)	Area under ROC curve (%, 95 % CI)
Sex	59.28	49.80	35.67	54.54 (52.47–56.61)
Age 65 and over	29.25	77.14	37.36	53.19 (51.31–55.08)
Current smoker	16.42	83.97	32.46	50.19 (48.60–51.79)
Depressive symptoms	18.69	92.18	52.38	55.44 (53.91–56.96)
Intermediate to high alcohol risk	6.26	94.44	34.85	50.35 (49.31–51.40)
Not physically active	31.40	72.90	35.57	52.15 (50.08–54.22)
BMI obese	39.36	74.41	41.82	56.88 (54.83–58.94)
Diabetes	10.74	92.10	38.94	51.42 (50.13–52.71)
Arthritis	41.05	79.07	47.58	60.06 (58.06–62.06)
Foot pain	35.16	91.70	66.18	63.43 (61.61–65.24)
Heel pain	7.63	98.06	64.77	52.85 (51.84–53.86)
Ball pain	10.17	98.00	70.37	54.09 (52.95–55.23)
Arch pain	9.24	98.00	68.32	53.62 (52.52–54.71)
Nail pain	0.67	99.75	55.56	50.21 (49.89–50.53)
Toe pain	12.72	97.25	68.35	54.98 (53.72–56.25)
Forefoot pain	15.53	96.38	66.67	55.95 (54.57–57.33)
Hindfoot pain	9.50	97.25	61.74	53.38 (52.25–54.50)
Podiatry use	18.26	91.71	50.54	54.99 (53.47–56.50)
Hip pain/stiffness	18.72	91.86	51.47	55.29 (53.74–56.84)
Back pain/stiffness	48.76	68.39	41.56	58.58 (56.48–60.67)
Hand pain/stiffness	25.88	88.13	50.12	57.00 (55.27–58.74)
Shoulder pain/stiffness	35.16	80.23	45.06	57.70 (55.75–59.64)
Knee pain/stiffness	25.76	86.99	47.56	56.38 (54.62–58.13)

Multivariable analysis was undertaken, with generalised linear models used to determine the RR of each of the factors in association with foot pain on most days in the last month compared to not having foot pain on most days. Four models were undertaken, the first using the reported prevalence of foot pain only and the second using the location of foot pain (Table 3). Models 3 and 4 repeated Models 1 and 2, respectively, but included the areas of joint pain variables (upper limb, lower limb, back pain) (Table 4). The factors associated with the presence of foot pain in 2008–2010 were: symptoms of depression, self-reported arthritis, BMI classified as obese, foot pain (general and specific areas) and reporting having experienced shoulder and/or hand pain.

**Table 3:** Relative risk of predictor variables associated with foot pain on most days in the past month (Model 1 and 2)

	Model 1		Model 2	
	RR (95 % CI)	*p*-value	RR (95 % CI)	*p*-value
Sex	1.14 (0.99–1.35)	0.146	1.10 (0.92–1.31)	0.307
Age 65 and over	1.16 (0.99–1.37)	0.073	1.13 (0.95–1.34)	0.163
Current smoker	1.22 (0.97–1.52)	0.084	1.12 (0.91–1.39)	0.289
Depressive symptoms	1.62 (1.32–1.99)	<0.001	1.67 (1.37–2.04)	<0.001
Intermediate to high alcohol risk	1.29 (0.94–1.77)	0.110	1.39 (1.02–1.90)	0.037
Not physically active	1.01 (0.84–1.21)	0.930	1.06 (0.89–1.26)	0.540
BMI obese	1.34 (1.12–1.59)	0.001	1.33 (1.12–1.59)	0.001
Diabetes	0.98 (0.80–1.21)	0.874	1.04 (0.83–1.29)	0.759
Arthritis	1.48 (1.27–1.72)	<0.001	1.50 (1.27–1.77)	<0.001
Podiatry use	1.15 (0.94–1.40)	0.189	1.19 (0.98–1.46)	0.082
Foot pain	2.19 (1.87–2.57)	<0.001		
Heel pain			1.41 (1.09–1.84)	0.010
Ball pain			1.39 (1.13–1.71)	0.002
Arch pain			1.12 (0.87–1.45)	0.386
Nail/Toe pain			1.23 (0.99–1.51)	0.056
Forefoot pain			1.51 (1.27–1.80)	<0.001
Hindfoot pain			1.37 (1.04–1.79)	0.024

**Table 4:** Relative risk of predictor variables associated with foot pain on most days in the past month (Model 3 and 4)

	Model 3		Model 4	
	RR (95 % CI)	*p*-value	RR (95 % CI)	*p*-value
Sex	1.13 (0.95–1.34)	0.176	1.09 (0.91–1.30)	0.349
Age 65 and over	1.18 (1.00–1.39)	0.056	1.16 (0.98–1.37)	0.085
Current smoker	1.23 (0.98–1.54)	0.074	1.15 (0.93–1.43)	0.183
Depressive symptoms	1.51 (1.21–1.89)	<0.001	1.55 (1.28–1.89)	<0.001
Intermediate to high alcohol risk	1.22 (0.85–1.74)	0.290	1.25 (0.87–1.79)	0.222
Not physically active	1.00 (0.83–1.20)	0.974	1.02 (0.86–1.22)	0.787
BMI obese	1.30 (1.08–1.56)	0.005	1.27 (1.07–1.51)	0.007
Diabetes	0.96 (0.78–1.19)	0.729	1.02 (0.81–1.28)	0.872
Arthritis	1.30 (1.09–1.54)	0.003	1.27 (1.06–1.53)	0.009
Podiatry use	1.17 (0.95–1.44)	0.139	1.21 (0.98–1.48)	0.073
Foot pain	1.96 (1.66–2.32)	<0.001		
Heel pain			1.44 (1.13–1.84)	0.004
Ball pain			1.41 (1.14–1.73)	0.001
Arch pain			1.18 (0.92–1.50)	0.187
Nail/Toe pain			1.12 (0.91–1.38)	0.296
Forefoot pain			1.43 (1.19–1.71)	<0.001
Hindfoot pain			1.21 (0.90–1.63)	0.209
Upper limb pain/stiffness	1.35 (1.09–1.68)	0.007	1.41 (1.14–1.75)	0.001
Lower limb pain/stiffness	1.05 (0.86–1.28)	0.639	1.06 (0.87–1.30)	0.545
Back pain/stiffness	1.16 (0.96–1.40)	0.116	1.21 (1.00–1.45)	0.048

## Discussion

The results of this study demonstrate that foot pain is a common condition within the general population. While there has been some recent work attempting to identify phenotypes using radiographic information for those with foot osteoarthritis [28] there has been little work, particularly across all age groups, attempting to identify the predictors of generalised foot pain. This study used a questionnaire and a set of clinical measures to collect specific data as part of a cohort study to determine whether these variables predicted the presence of foot pain at a later stage.

The variables with the highest positive predictive values: BMI, depression, areas of foot pain, self-reported arthritis and podiatry use were those most strongly associated with the occurrence of foot pain on most days in the past month in the multivariable model and indicate that there are likely to be specific characteristics concomitant with foot pain, even though these variables have a relatively low ability to predict the occurrence of foot pain (the highest area under the ROC curve being 70.37 for ball of the foot pain). Addressing these associated factors are consequently still likely to impact on the overall prevalence of foot pain.

Those with symptoms of depression and classified as obese according to their BMI in 2004–2006 were at higher risk of reporting foot pain in 2008–2010. There have been strong cross-sectional associations demonstrated between foot pain and BMI [5, 29], which is likely to be biomechanical in nature due to increased pressure under the feet. Gay et al. [30] have also demonstrated, using longitudinal data, that foot joint pain was highly prevalent among middle aged women and that a high BMI was predictive of foot pain, independent of age. In this study, the probability of being obese was 41.8 % if participants reported foot pain on most days in the last month and when examined in the multivariable model, the risk of reporting a high BMI remained. However, recent work has also identified that fat mass and the association with biochemical markers may play a greater role in the development of foot pain than BMI [29, 31, 32] and further examination of these issues may be important over the longer term.

An association between foot pain and depression has previously been shown [32] and the development of major depressive disorders may occur as a consequence of inflammation [33]. Inflammatory markers are associated with fat mass and thus may impact on the development of depression. There is also a relationship between obesity itself and depression [34]. These factors combined are likely to have an effect on the association between depression and foot pain.

It is not surprising that those reporting arthritis or foot pain in 2004–2006, whether this is general foot pain or by location, were at a higher risk of reporting foot pain in 2008–2010. Multiple joint problems such as those that occur with arthritis have been shown to be more common than single joint problems [18]. Hill et al. [3] have also demonstrated that foot pain in the North West Adelaide cohort is also associated with reports of pain in other joints. Gill et al. [19] demonstrated that shoulder pain was associated with pain in other joint areas, which may be as a result of arthritis. The apparent existence of foot pain over a period of time provides an indication of chronicity and burden. Chronic musculoskeletal pain is associated with longer-term declines in overall health and physical mobility [35]. Efforts should be made to determine if earlier intervention can limit these declines, which are not only characterised by painful feet, but by the negative sequelae associated with physical inactivity [36].

There are three main limitations of this study. Firstly, the foot pain questions were non-specific, lacked a clinical diagnosis, and did not involve objective measures such as x-rays. Secondly, the sample has been obtained from the metropolitan area of a city in Australia, and thus the factors predictive of foot pain may be different in other populations. Thirdly, the focus of the study is on predictive factors, and as such causation cannot be inferred. Strengths of this study are the use of a longitudinal cohort with questions relating to foot pain asked at two time points, the level of detail collected on the location of foot pain and the data available over a 6–7 year time period. More than 2000 participants provided responses to the foot pain questions in 2004–2006 and 2008–2010 and a broad range of covariates were available for analysis. To our knowledge there are no previous longitudinal studies of foot pain from a population-based sample with the same breadth of covariates conducted in Australia.

## Conclusion

Foot pain affects a significant proportion of the population over a period of time. Those reporting depression, arthritis, previous foot pain, upper limb pain and a high BMI were at greater risk of reporting foot pain on most days in the last month. However, the sensitivity, specificity and positive predictive values of variables to predict the presence of foot pain varied greatly. Those with the greatest risk of having foot pain were those with arthritis and previous foot pain, which indicates a high level of chronicity and subsequent burden on the health system. Addressing BMI, depression and general joint health with the use of targeted messages to highlight the impact of foot pain on daily functioning may be of benefit in order to reduce the burden of foot pain on the population.

## Abbreviations

BMI, body mass index; CATI, computer assisted telephone interviewing; CES-D, Centre for Epidemiological Studies in Depression questionnaire; CI, confidence interval; NWAHS, North West Adelaide Health Study; ROC, receiver operating characteristic; RR, relative risk
